# AlphaFold2: A Role for Disordered Protein/Region Prediction?

**DOI:** 10.3390/ijms23094591

**Published:** 2022-04-21

**Authors:** Carter J. Wilson, Wing-Yiu Choy, Mikko Karttunen

**Affiliations:** 1 Department of Mathematics, The University of Western Ontario, 1151 Richmond Street, London, ON N6A 5B7, Canada; cwils256@uwo.ca; 2 Centre for Advanced Materials and Biomaterials Research, The University of Western Ontario, 1151 Richmond Street, London, ON N6A 5B7, Canada; 3 Department of Biochemistry, The University of Western Ontario, 1151 Richmond Street, London, ON N6A 5C1, Canada; 4 Department of Physics and Astronomy, The University of Western Ontario, 1151 Richmond Street, London, ON N6A 5B7, Canada; 5 Department of Chemistry, The University of Western Ontario, 1151 Richmond Street, London, ON N6A 3K7, Canada

**Keywords:** AlphaFold2, disordered proteins, IDPs/IDRs, machine-learning, biophysics, structural bioinformatics, molecular dynamics, simulation

## Abstract

The development of AlphaFold2 marked a paradigm-shift in the structural biology community. Herein, we assess the ability of AlphaFold2 to predict disordered regions against traditional sequence-based disorder predictors. We find that AlphaFold2 performs well at discriminating disordered regions, but also note that the disorder predictor one constructs from an AlphaFold2 structure determines accuracy. In particular, a naïve, but non-trivial assumption that residues assigned to helices, strands, and H-bond stabilized turns are likely ordered and all other residues are disordered results in a dramatic overestimation in disorder; conversely, the predicted local distance difference test (pLDDT) provides an excellent measure of residue-wise disorder. Furthermore, by employing molecular dynamics (MD) simulations, we note an interesting relationship between the pLDDT and secondary structure, that may explain our observations and suggests a broader application of the pLDDT for characterizing the local dynamics of intrinsically disordered proteins and regions (IDPs/IDRs).

## 1. Introduction

Predicting the three-dimensional structure of a protein from its primary amino acid sequence is a grand challenge in molecular structural biology dating back to the late 1950’s [1,2]. About a year and a half ago, AlphaFold2 (AF2), a deep-learning program, provided a paradigm-shift in this problem [3]. Not only did it outperform all other groups at the 14th Critical Assessment of protein Structure Prediction (CASP14) [3], but it did so with astonishing accuracy and a large margin. Consequently, this breakthrough has caused enthusiasm in several related fields, including drug development [4].

The full problem of protein folding is, however, multi-faceted, and despite AlphaFold’s stellar success, many problems and open questions remain. As has already been pointed out by several authors [5,6,7,8,9], dynamics of protein folding remains a formidable problem; prediction of the folding pathways, effects of mutations, the solution environment, aggregation and, as a very particular category, intrinsically disordered proteins and regions (IDPs/IDRs).

IDPs remain a major challenge since they are almost entirely devoid of native structure and because they function primarily as conformational ensembles [10,11,12,13,14,15,16,17] with folding free energy landscapes that are relatively flat [18,19,20]. This is a direct consequence of their amino acid sequences [21,22,23], in particular the enrichment of disorder-promoting residues over and above order-promoting ones [24,25,26,27]. The application of AF2 to the prediction of IDRs and IDPs has only briefly been discussed in the literature [6,7,8,28], and its performance against multiple traditional predictor methods is currently absent.

In light of the recent publication of the critical assessment of protein intrinsic disorder (CAID) benchmark [29], detailing the performance of over three dozen sequence-based disorder predictors and their datasets, we saw an excellent opportunity to benchmark AF2. Herein, we compare the performance of AF2 to the top performing sequence-based disorder predictors as determined at CAID. Importantly, while we find AlphaFold2 to perform exceptionally well on disorder identification; we also note that the disorder predictor one constructs from an AlphaFold2 structure determines accuracy. Specifically, a naïve, non-trivial assumption that the structure assignment provided by DSSP [30], the primary method for assigning secondary structure based on protein geometry, can be used for the determination of disordered regions, leads to a dramatic overestimation in disorder content and represents a potential pitfall for researchers who are less familiar with IDPs and structural prediction methods.

The predicted local distance difference test (pLDDT), which is correlated to the confidence of the structure prediction, provides a better metric for identifying ordered and disordered regions. Furthermore, we find that traditional predictors are capable of outperforming AF2 in disorder prediction even when the pLDDT is used. We also show how secondary structure and pLDDT scores are interestingly related, providing a potential explanation for the observed performance discrepancy and highlight a link between local protein dynamics and the pLDDT using a well characterized IDP and MD simulations.

## 2. Methodology

### 2.1. Dataset Generation

Two datasets were used in this work, DisProt and DisProt-PDB, derived from the DisProt database [31]. Both reference sets are based on the CAID benchmark dataset and are composed of 475 targets, annotated between June and November 2018 (DisProt release 2018_11). Note that this is less than the 646 targets used at CAID because AF2 predicted structures do not exist for some sequences. In the DisProt reference set, all residues not labeled as disordered (1) are labeled as ordered (0). We would like to note that such a definition has significant limitations and the conclusions we draw herein are principally based on the DisProt-PDB dataset. Figures and tables based on the DisProt set are found in Supplemental Information and care should be taken when drawing conclusions from them. Our decision to include them here is simply for completeness. The DisProt-PDB reference set, on the other hand, only annotates residues for which some experimental data are available; either a PDB structure that suggests a residue to be ordered or experimental findings, catalogued in DisProt, which suggest a residue to be disordered. Note that if a conflict arises between a DisProt entry suggesting disorder and a PDB structure suggesting order, a disordered assignment is made. All residues not covered by PDB structures or DisProt annotations are masked and were excluded from analysis. As a result, the DisProt-PDB dataset contains no ’uncertain’ residues. All residues considered in this set have either a DisProt annotation, based on prior literature, or belong to a PDB structure. We note that the EMBL/AF2 database contains some structures that are present in the dataset. The degree to which this improves the performance of AF2 is not easily measured; however, it is our belief the impact to be small. Additional details pertaining to dataset construction are provided in Supplementary Information and the full list of proteins, structures, and combined disorder data are available at https://github.com/SoftSimu/AlphaFoldDisorderData (accessed on 21 September 2021).

AF2 structures were downloaded from the EMBL database (https://alphafold.ebi.ac.uk/, accessed on 21 September 2021) and run using DSSP [30] to assign secondary structure. We assume that residues belonging to helices, strands, or H-bond stabilized turns are ordered (0) and all other residues are disordered (1). We refer to this as the näive DSSP predictor or DSSPp for short.

We also collected pLDDT values for each structure. Every residue in an AF2 structure is assigned a value, scaled between 0 and 100, which predicts the Cα local distance difference test (lDDT) [3,28,32] score of a model; in short, this metric captures the residue-wise confidence of an AF2 model. We transform this value according to the equation,

(1) tpLDDT=1−pLDDT/100,

as suggested by Tunyasuvunakool et al. [28], giving us a pLDDT-based predictor of disorder, where 1 is disordered and 0 is ordered. We refer to this prediction method as the transformed pLDDT or tpLD for short.

We can discretize this pLDDT predictor by classifying a residue with a pLDDT score ≥*n* as ordered (0) and disordered (1) otherwise; we use pLDDT${}_{n}$ (or pLD${}_{n}$ for short), to indicate this binary predictor. Thresholds for *n* were chosen based on the Matthews correlation coefficient (MCC), which has been documented to be an excellent metric for assessing the accuracy of binary classifiers [33] and was the approach used at CAID [29]. Notice this gives us two predictors: (1) a continuous predictor (tpLDDT) where a residue’s degree of disorderedness is captured, and (2) a discrete predictor (pLD${}_{n}$) where a residue is either disordered or ordered depending on the pLDDT and chosen threshold (*n*). The CAID dataset contains predictions made by three dozen predictors. We selected the top 10 performing on the DisProt and DisProt-PDB giving a combined non-redundant set of 11 (fIDPnn [34], SPOT-Disorder2 [35], RawMSA [36], fIDPlr [34], PreDisorder [37], AUCpreD [38], SPOT-Disorder1 [39], SPOT-Disorder-Single (SPOT-Disorder-S) [40], DisoMine [41], AUCpreD-np [38] and ESpritz-D [42]). The sequence predictors provide a score between 0 and 1, inclusive, as well as a binary disorder/order assignment. No modification to the classification thresholds for these predictors was attempted. Descriptions of disorder prediction methods are provided in the Supplementary Information of the original CAID paper [29]. For two vectors, *v* and *w*, we compute the RMSD as

(2) $$\mathrm{RMSD}=\sqrt{\frac{1}{m}\sum_{i=1}^{m}{\left|v_{i}-w_{i}\right|}^{2}},$$

where *m* is the number of elements (residues) in each vector (protein), *v* and *w*. Given binary vectors, a random predictor has an RMSD of ∼0.7 on a uniform dataset. Receiver operating characteristic (ROC), area under the curve (AUC), precision–recall, F${}_{1}$-score, and correlation analysis were all performed using scikit-learn [43], and kernel density estimate (KDE) analysis was performed in seaborn [44]. Descriptions of statistical methods are provided in Supplementary Information.

### 2.2. Nrf2 Structure Generation

We used ColabFold [45] to generate both Neh4 and Neh5 structures, our model IDP systems. Two approaches were used: the first was to consider the peptide sequences used in our previous work [46,47], specifically ^111^SDALYFDDCMQLLAQTFPFVDDN^133^ and ^180^MQQDIEQVWEELLSIPELQCLNIENDKLVE^209^. These are the Neh4 and Neh5 domains, respectively. The second approach was to consider the more realistic construct that includes the linker ^106^AHIPKSDALYFDDCMQLLAQTFPFVDDNEVSSATFQSLVPDIPGHIESPVFIATNQAQSPETSVAQVAPVDLDGMQQDIEQVWEELLSIPELQCLNIENDKLVETTMVP^214^ and extract the local structures comprising the domains. ColabFold generates five ranked structures per sequence giving rise to three pools of structures. Alignment of the structures within the Neh4 and Neh5 pools showed excellent agreement and we opted to simply consider the top-ranked structures in each pool, denoted Neh4 (P) and Neh5 (P). Alignment of these peptide structures to the longer construct suggests good agreement; however, there were some constructs with structural differences. We consider the construct that was the most heterogeneous with respect to the smaller peptides and extracted the local Neh4 and Neh5 structures, denoted Neh4 (C) and Neh5 (C).

### 2.3. Molecular Dynamics Simulations

The MD simulation protocols for the two force fields were almost identical, the primary difference was that the simulations using the Amber-99SB*-ILDNP [48,49,50] force field were performed at 310 K with the TIP3P water model [51] while the Amber99SB-*disp* [52] simulations were performed at 298.15 K with the TIP4P-*disp* water model [52]. Note that the Amber-99SB*-ILDNP simulations were taken from our previous work [46] while the Amber-99SB*-*disp* runs were new to the work discussed herein. In both cases, the steepest descent algorithm was utilized for energy minimization, temperature was maintained using the Parrinello–Donadio–Bussi velocity rescaling method [53] with a 1.0 ps coupling time and pressure were maintained using the Parrinello–Rahman barostat [54] at 1 bar with a coupling time of 5.0 ps. The simulation time step was 2.0 fs. Long-range electrostatic interactions were calculated using the particle-mesh Ewald (PME) method [55] with a Fourier spacing of 0.12 nm and a real-space cut-off of 1.0 nm; the Lennard–Jones interactions were computed with a 1.2 nm cut-off. H-bonds were constrained using the LINear Constraint Solver (P-LINCS) [56]. K${}^{+}$ or Cl${}^{-}$ ions were added to neutralize excess charge, i.e., overall charge neutrality was always preserved. Each simulation was performed in quadruplicate for 3 μs, totalling 12 μs of simulation time for each force field–protein combination.

## 3. Results

### 3.1. pLDDT Performs Better Than Conventional Predictors and a Näive Use of DSSP for Disorder Identification

**Figure 1:**
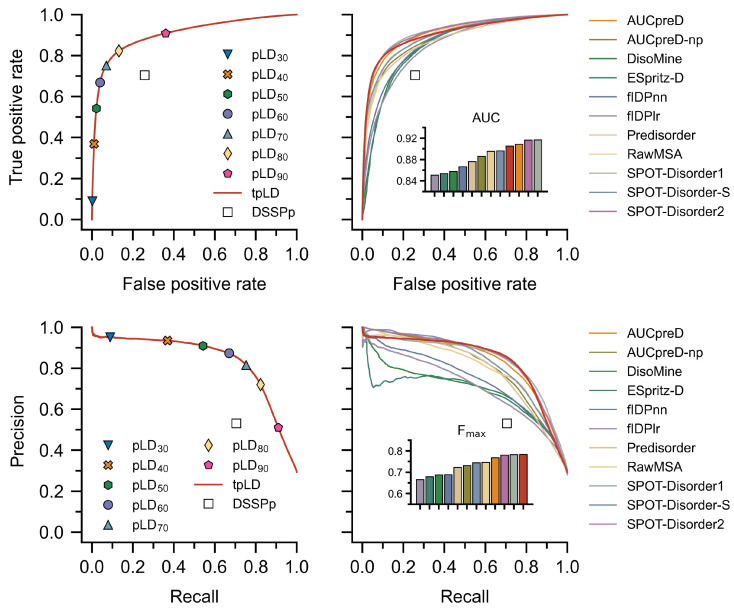
Receiver operating characteristic (ROC) curves (**top**) and precision–recall (**bottom**) are depicted for various predictors calculated per residue on the DisProt-PDB dataset. Note that a ROC curve captures the probability of true and false positives at all thresholds, where an ideal predictor will have an area under the curve (AUC) equal to 1. Further note that a precision–recall curve captures the trade-off between precision and recall; again, in the ideal case the harmonic mean of the precision and recall (F${}_{\max}$) will be equal to 1; bar colors correspond to the legend, red denotes tpLD. In all cases the tpLD (Equation (1)) and various discrete pLD${}_{n}$ predictors are indicated alongside DSSPp. The tpLD predictor resulted in one of the highest AUC values and the highest F${}_{\max}$ on the DisProt-PDB dataset. pLDDT is abbreviated as pLD for plotting purposes.

**Figure 2:**
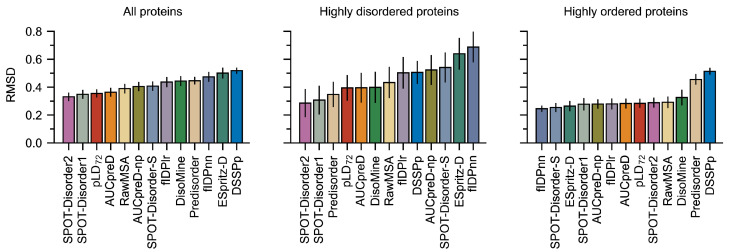
Average RMSD (Equation (2)) values calculated for the DisProt-PDB datasets using various prediction methods calculated per protein. Proteins were assigned to classes (highly disordered i.e., >90% disorder and highly ordered i.e., <10% disorder) based on datasets. Bootstrapping—that is, sampling with replacement—was used to compute averages and estimate errors with 10,000 samples of size 60. pLD${}_{72}$ resulted in lower RMSD values on the DisProt-PDB dataset compared to DSSPp. pLDDT is abbreviated as pLD for plotting purposes.

**Figure 3:**
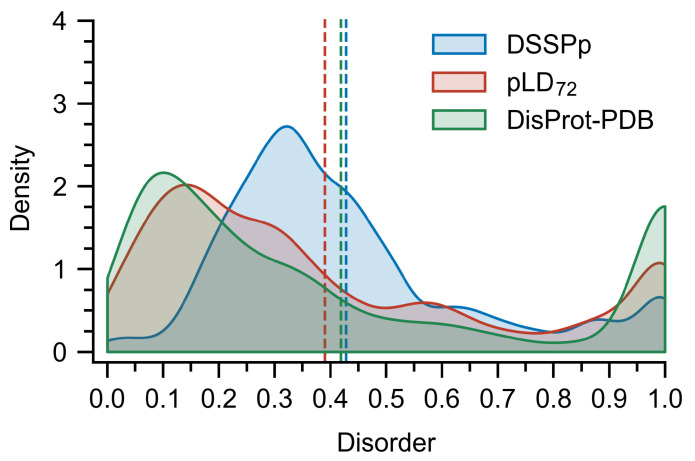
Distribution of disorder content per protein in the DisProt-PDB dataset depicted alongside the distributions predicted by pLD${}_{72}$ and DSSPp. Bin-widths were set at 0.5 and bootstrapping that is, sampling with replacement, was used to compute the distributions and average values (vertical dashed lines) with 10,000 samples of size 60. Close agreement between the experiment and pLD${}_{72}$ is evident, conversely, DSSPp predicted a higher disorder content. pLDDT is abbreviated pLD for plotting purposes.

Improved performance with tpLD (Equation (1)) over and against conventional predictors and a näive application of DSSPp is evidenced by the ROC curves and AUC values (Figure 1 and Figure S1), as well as the precision–recall (PR) curves and F${}_{\max}$ values (Figure 1 and Figure S1) on both the DisProt-PDB and DisProt datasets (Tables S1 and S2). Thresholds for the binary pLD${}_{n}$ predictor were selected based on the Matthews correlation coefficients, which gave values of 76 and 68 for the DisProt and DisProt-PDB datasets respectively (Tables S3 and S4). We refer to these discrete predictors as pLD${}_{76}$ and pLD${}_{68}$. Unsurprisingly, these values agree with the minimum distance from the ROC curve to the top left of the plot (i.e., (0, 1)) (Figure 1). The difference between these two values undoubtedly stems from the nature of the underlying datasets: while DisProt-PDB contains no uncertain residues, DisProt does. For analysis purposes, we opted to use a combined pLDDT metric, denoted pLD${}_{72}$, which is the mean of these two. Data using multiple pLDDT values are provided in Tables S1 and S2. RMSD (Equation (2)) calculations comparing DSSPp and pLD${}_{72}$ demonstrate improved performance for all protein classes, including highly disordered (i.e., >95%) and highly ordered (i.e., <10%), irrespective of dataset (Figure 2 and Figure S2). We note that overall RMSD values are on average lower for the DisProt-PDB dataset, again likely a result of it lacking “uncertain” residues—residues for which no PDB or experimental data exists. Shifts towards lower RMSD irrespective of dataset, or protein length and disorder content, are also evident for pLD${}_{72}$ (Figures S4 and S5). A regression analysis revealed stronger correlations between pLD${}_{72}$ and the traditional disorder predictors with respect to residue-wise disorder RMSD when compared with DSSPp (Figures S6–S9). Considering global disorder content prediction, we find that on the DisProt dataset pLD${}_{72}$ shows slightly better performance than DSSPp with a lower mean and a more accurate distribution; however, we note that both methods significantly overestimate disorder content (Figure 3 and Figure S3). On the DisProt-PDB dataset, closer agreement between pLD${}_{72}$ and DSSPp is evident based on the mean with both methods returning values similar to experiment. The two distributions are, however, notably different. While that produced by pLD${}_{72}$ has a peak around 0.15, in close agreement with the experiment, the peak in the distribution produced by DSSPp is larger and shifted to a higher value around 0.3. This is all to say that a näive application of DSSP for the prediction of disordered and ordered regions for AF2 structures, specifically the assumption that helical and strand regions are ordered, and coiled regions are unstructured, leads to poorer prediction (i.e., higher RMSD, lower AUC, and higher F${}_{\max}$) of disordered regions and an overestimation in disorder content.

### 3.2. Sequence Predictors Can Still Outperform AlphaFold2 on Disorder Prediction

Comparing the pLDDT-based and DSSPp predictors to various sequence-based predictors revealed performance differences amongst the methods. Notably, tpLD (Equation (1)) performed exceptionally well on the DisProt-PDB dataset posting the largest F${}_{\max}$ (0.784) and one of the largest AUC (0.905) values of the methods considered (Figure 1, Tables S1 and S3). This was also evidenced by pLD${}_{72}$, which had the highest MCC (0.701) (Table S1) and one of the lowest RMSD values (Figure 2) on the DisProt-PDB dataset. Unsurprisingly, on the DisProt dataset, both tpLD (Equation (1)) and DSSPp performed significantly worse and were readily outperformed by the other predictor methods, in particular fIDPnn (F${}_{\max}$: 0.357 (DSSPp), 0.429 (tpLD), 0.457 (fIDPnn); AUC: 0.635 (DSSPp), 0.731 (tpLD), 0.794 (fIDPnn)), which outperformed all other predictors, as evidenced by the ROC, PR, and RMSD analyses. We note that with respect to MCC, pLD${}_{72}$ still performed well on both the DisProt and DisProt-PDB datasets achieving scores of 0.310 and 0.697, respectively (Tables S1 and S2). In agreement with the CAID results, we found that SPOT-Disorder2, fIDPnn, RawMSA, and AUCpreD all performed exceptionally well (Figure 1 and Figure S1, Tables S3 and S4) [29].

### 3.3. Secondary Structure Codons (SSC) Suggests Relationships between the pLDDT and Secondary Structure

**Figure 4:**
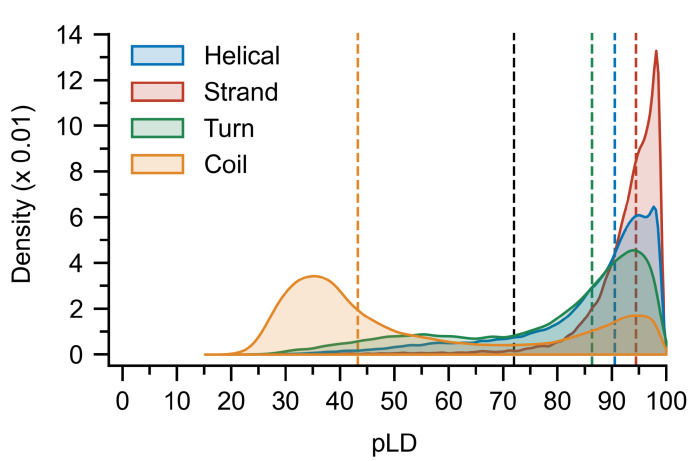
Distribution of pLDDT values per residue calculated for each secondary structure class. Bin-widths were set at 0.5 and bootstrapping, that is, sampling with replacement, was used to compute the distributions and mean values (colored vertical dashed lines; black dashed line represents pLD${}_{72}$) with 10,000 samples of size 500. A bimodal distribution is evident for the coil structures, and while strand, helical, and turn regions are on average assigned to high pLDDT values, residues belonging to each can sample much lower values. pLDDT is abbreviated pLD for plotting purposes.

In order to explain the discrepancy between the pLDDT-based and DSSP predictors with respect to local and global disorder prediction, we considered how pLDDT values were assigned to the secondary structures. Kernel density estimates (KDE) of the distribution of pLDDT values sampled over all residues revealed a strong left-skew for all but the coil secondary structure, which exhibited a right-skewed bimodal distribution with peaks around 94 and 35 (Figure 4). Residues assigned to β-strand and β-bridge structures are the most likely to be assigned to large pLDDT values, followed by helical and H-bond stabilized turns. To provide a more detailed picture of the distributions, we introduce the concept of a secondary structure codon (SSC), a triplet describing the local secondary structure at a given residue. Analysis of the distributions of pLDDT values for each SSC revealed that residues predicted to belong to both the ends (HHC/CHH/HHT/THH) and middle (HHH) of helices can have pLDDT values <50 (Figure S10), this was not observed for residues belonging to the middle (EEE) and ends of β-strands (EEC/CEE/EET/TEE) (Figure S11). For highly coiled residues (CCC/CCT/TCC) and several turn residues (CTT/TTC), both high (>80) and low (<50) pLDDT values were observed (Figures S12 and S13).

### 3.4. Nrf2: A Case Study

**Figure 5:**
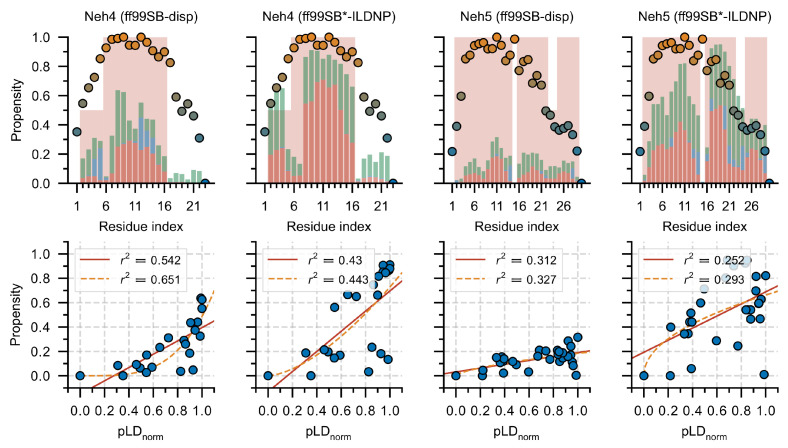
Secondary structure of ensembles versus AF2. **Top**: Secondary structure was computed from molecular simulation (red = α-helix, $3_{10}$-helix or π-helix; blue = β-strand or β-bridge; and green = turn). The red background color depicts the AF2 predicted secondary structure propensities, no strand/turn content was predicted. **Bottom**: Min–max normalized pLDDT values (${\mathrm{pLD}}_{\mathrm{norm}}$) are plotted (circles) with colors ranging from 0 to 1 (orange implies ${\mathrm{pLD}}_{\mathrm{norm}}=1$ and blue implies ${\mathrm{pLD}}_{\mathrm{norm}}=0$). We plot correlations between the total secondary structure propensity computed from MD simulations and the ${\mathrm{pLD}}_{\mathrm{norm}}$, and fit the data to a line (red) or a power law (orange). pLDDT is abbreviated pLD for plotting purposes.

**Figure 6:**
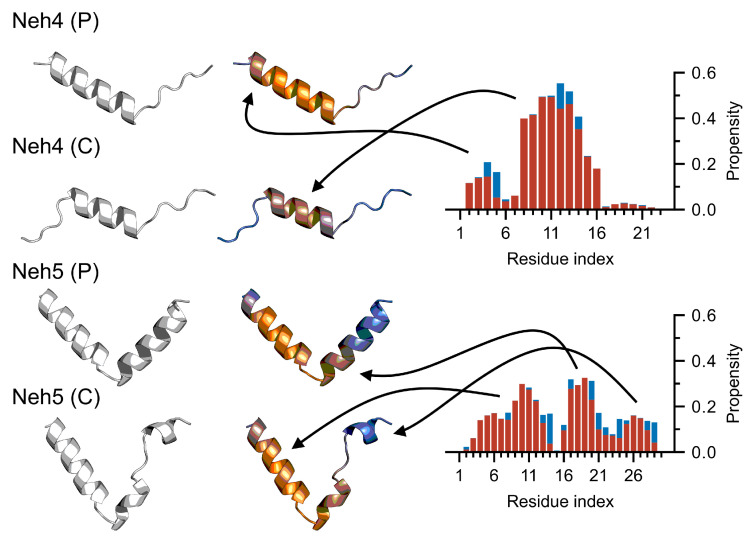
AF2 predicted structures correlate with simulated secondary structure. We consider the peptide (i.e., Neh4/5 (P)) and construct (i.e., Neh4/5 (C)) structures predicted from AF2, without a colormap and with a pLDDT colormap scaled between 70 and 100 (i.e., blue implies pLDDT=70 and orange implies pLDDT=100). Note how the coloring of the structures provides non-trivial insights that are undetectable without it. These are depicted alongside the average secondary structure computed using both the ff99SB*-ILDNP and ff99SB-*disp* simulations (red = α-helix, $3_{10}$-helix or π-helix; blue = β-strand or β-bridge). Note that arrows indicate corresponding regions between AF2 structures (**left**) and structural propensities computed from MD simulations (**right**).

Nrf2 (nuclear factor erythroid 2-related factor 2) is a partially disordered transcription factor [47,57] and is the master regulator of the cellular anti-oxidative response. Within the multi-domain Nrf2 protein, two transactivation domains, namely Neh4 and Neh5, are responsible for binding the transcriptional adaptor zinc-binding domains, TAZ1 and TAZ2, of CBP; references [58,59]; previous work has elucidated the free-state ensembles of Neh4 and Neh5 using both MD simulations and circular dichroism [46]. We consider the AF2 predicted structures of the Neh4 and Neh5 peptides (Neh4 (P) and Neh5 (P)) and the structures predicted for Neh4/5 within a larger construct (Neh4 (C) and Neh5 (C)). Comparison of the secondary structures determined from the AF2 predictions and simulated ensembles suggested relatively good agreement; regions of low helical propensity in the ensemble corresponded to lower helical propensity in the AF2 structures, and the converse was also true (Figure 5). There also appeared to be some agreement between pLDDT and secondary structure; however, these correlations were weak (Figure 5) and depended strongly on the system considered (Neh4 vs. Neh5). We also overlaid the pLDDT with the predicted structures seeking to assess the potential for additional insights. Immediately evident was the heterogeneity in the predicted structures when considering the peptide and the larger construct. Notably, the differences in the structure occurred precisely where the pLDDT was lower (e.g., the N-terminal of the Neh4 (P) that was not present in the Neh4 (C) and the C-terminal helix in Neh5 (P) that was split in Neh 5 (C)). The pLDDT and heterogeneity of the structures in particular with Neh5, agreed closely with the observed secondary structure from the ensembles (Figure 5 and Figure 6); specifically, the triple helix, with a hard break at I14-P15 and a transient break from N22–E24. These structural dynamics—that is the exchange between a large and a small helix in the C-termini of Neh5—appeared to be captured explicitly by the pLDDT and implicitly by the heterogeneity of the AF2 structures.

## 4. Discussion

AF2 has been a paradigm-shift in structural biology, providing a tentative solution to the protein folding problem that has persisted over half a century [1]. Since the time that problem was posed by Perutz and Kendrew, a new class of proteins, intrinsically disordered proteins, has been discovered and IDPs have become the focus of much study [10,11,13,14,60,61]. Over the past two decades, much effort has been devoted to developing methods for identifying disordered regions given the primary sequence of a protein [29,62,63,64,65,66]. Herein, we assess the applicability of AF2 to this problem.

We find (and strongly stress) that simply inferring a residue in an AF2 structure assigned by DSSP to a helical, strand, or H-bond stabilized turn is ordered, and otherwise is disordered, results in an overestimation of disorder content and a poor prediction of disordered regions. While this may seem like a trivial observation, the abundance of AF2 structures generated for disordered proteins has made such a pitfall increasingly likely for researchers who are less familiar with IDPs and structural prediction methods. Instead, employing the pLDDT, a measure of the expected position error at a given residue and originally purposed to assess the residue-wise structural confidence, provides a much more accurate metric for determining global and local disorder content. Using the pLDDT as a disorder predictor metric, we observe impressive performance on the DisProt-PDB dataset when compared to conventional disorder predictors (Figure 1). We here note the work by Akdel et al. [8], who found that, in addition to the pLDDT, the solvent accessible surface area of an AF2 structure provides another strong predictor of disorder. Similar to our 2021 benchmark published in bioRxiv [67], this was recently extended by Piovesan et al. [68], wherein a combined RSA-pLDDT metric for assessing IDP binding was considered.

Secondary structure and global disorder analyses point to a potential root of the prediction discrepancy between pLDDT and DSSP; simply put, for AF2, not all secondary structures are created equal. AF2 will readily assign a coiled geometry and a high pLDDT value to the same residue, and conversely assign low pLDDT values to structured regions (Figure 4). While a näive DSSP predictor assumes that coils and bends are disordered while helices, strands, and turns are ordered, a pLDDT predictor captures the biophysical reality that a coil may be more "ordered" and a helix more "disordered" for certain residues in certain proteins. It is this former case that likely results in the improved performance observed for pLDDT and underscores the importance of the nuance provided by this metric for disordered protein prediction. It also opens the door to another interesting question: is the conclusion to be drawn from two helices *A* and *B* of comparable geometry with significantly different average pLDDT (${\mathrm{pLDDT}}_{A}<{\mathrm{pLDDT}}_{B}$) simply that *A* is less likely to be “real”, or is it that both helices exist, however, *A* exists transiently?

The above question alludes to a second problem associated with IDP prediction, namely predicting the structural dynamics and transitions (i.e., order-to-disorder, disorder-to-order, disorder-to-disorder) that an IDP may undergo [62,69]. In light of the secondary structure analysis, the pLDDT may be just such a means for extracting this information, namely the transientness of secondary structures, their potential for transition upon binding and their functional importance. A helix with a low pLDDT may be more transient (i.e., existing frequently in a disordered, unfolded state) than a helix with a high pLDDT and conversely, a coiled region with a high pLDDT, may suggest a disorder–order transition and/or its conserved role in some biophysical interaction. The strength of AF2 as a predictor is that both a pLDDT score and a three-dimensional structure are provided, allowing for more comprehensive insights into an IDPs structure and dynamics. This is anecdotally evidenced by Nrf2, where considering the structure alone presents an incomplete story, that is quite literally colored in by the pLDDT, revealing something about the transientness of the C-terminal helix of Neh5. This hypothesis, pertaining to the relationship between the pLDDT and the structural transitions of IDRs, originally proposed in our 2021 pre-print [67], has been further substantiated by the findings of an impressive study by Alderson et al. [70] that systematically compared both NMR and AF2 data.

While the significance of this insight is buffeted by the unrealistically high helical content predicted by AF2, it appears to suggest that continued research into the pLDDT and heterogeneity of AF2 predicted structures may provide novel insights. We reiterate that, by their very nature, IDPs exhibit a high degree of conformational flexibility, allowing them to interact with multiple binding partners in a variety of ways [71,72,73,74,75,76,77,78]. While it is the case that a single, static, AF2 structure cannot adequately describe the totality of an often large conformational ensembles [13,14,15], the ability of the program to predict with relatively high accuracy the location of disordered regions is nonetheless impressive, and refinement of the training set to account for more accurate disordered structures could further improve performance. In addition, thorough analysis of the pLDDT score as it relates to structural transientness, as well as the local function and dynamics of IDP motifs, may further enhance the utility of AF2 to the IDP community.

While experimental NMR [47,79,80,81,82,83,84,85,86,87], and high-quality molecular simulations [46,88,89,90,91,92,93,94,95,96,97,98] are some of the most accurate methods for determining the (dis)ordered nature and dynamics of proteins, fast and computationally efficient methods play an important role. Unlike conventional predictors however, AF2 supplies both a pLDDT score, that can provide an accurate prediction of protein disorder, in addition to a three-dimensional structure, and when taken in tandem, these appear to provide insights into the underlying local dynamics (i.e., disorder–order transition) of disordered protein regions.

## 5. Conclusions

In this study, we assessed the ability of AF2 to predict disordered protein regions. We benchmark the program on two datasets developed for CAID [29], and find it to perform quite well, exceeding the performance of 11 traditional predictors on the DisProt-PDB dataset. Furthermore, we observe that the pLDDT score assigned to each residue by AF2 provides an impressive metric for assessing disorder, far surpassing a näive, but by no means, non-trivial application of DSSP for researchers who are less familiar with IDPs and structural prediction methods. Our analysis, in particular that of Nrf2, also suggests a novel link between secondary structure transience and the pLDDT score, intimating that continued research into this metric may reveal a connection to the local dynamics of disordered proteins.

## Data Availability

Additional details pertaining to dataset construction are provided in Supplementary Information and the full list of proteins, structures and combined disorder data are available at https://github.com/SoftSimu/AlphaFoldDisorderData (accessed on 21 September 2021).

## Supplementary Materials

The following supporting information can be downloaded at: https://www.mdpi.com/article/10.3390/ijms23094591/s1.
