# Supplementary material for: AlphaFold2: A Role for Disordered Protein/Region Prediction?
Source: Int J Mol Sci. 2022 Apr 21;23(9):4591. doi: 10.3390/ijms23094591 (PMC9104326; doi:10.3390/ijms23094591)
Supplement: Supplementary file 1 [file ijms-23-04591-s001.zip › ijms-1677250-supplementary.pdf]

Supplementary Information: AlphaFold2: A Role for Disordered Protein/Region Prediction?

Carter J. Wilson,[†,‡] Wing-Yiu Choy,[*,¶] and Mikko Karttunen[*,§,‖,‡]

†*Department of Mathematics, The University of Western Ontario, 1151 Richmond Street, Canada, N6A 5B7*

‡*Centre for Advanced Materials and Biomaterials Research, The University of Western Ontario, 1151 Richmond Street, London, Ontario, Canada, N6A 5B7*

¶*Department of Biochemistry, The University of Western Ontario, 1151 Richmond Street, Canada, N6A 5C1*

§*Department of Chemistry, The University of Western Ontario, 1151 Richmond Street, Canada, N6A 3K7*

‖*Department of Physics and Astronomy, The University of Western Ontario, 1151 Richmond Street, Canada, N6A 5B7*

E-mail: jchoy4@uwo.ca; mkarttu@uwo.ca

Supplemental Methods

Dataset generation

$$P = \begin{bmatrix} A & M & K & L & A & T & E & T & G & E & A & H & L & K & A & M \\ - & - & 1 & 1 & 1 & 1 & 1 & - & - & - & - & - & - & - & - & - \\ - & - & - & - & - & - & S & S & S & S & S & S & S & - & - & - \\ 0 & 0 & 1 & 1 & 1 & 1 & 1 & 0 & 0 & 0 & 0 & 0 & 0 & 0 & 0 & 0 \\ - & - & 1 & 1 & 1 & 1 & 1 & 0 & 0 & 0 & 0 & 0 & 0 & - & - & - \end{bmatrix}$$

P represents a single protein and some of the data used in the analysis. The first row is the amino acid sequence, the second row is the DisProt annotation (i.e. 1 = disordered, 0 = ordered, - = no data), the third row is the PDB annotation (i.e. - = no structural data present, S = structural data present), the fourth row is the DisProt dataset entry for this protein, the fifth row is the DisProt-PDB dataset entry for this protein. Note how residues with no DisProt data are assumed to be structured in the DisProt dataset entry while only residues for which either PDB or DisProt or both is available are included in the DisProt-PDB dataset entry. Also note that the residue 6 in the middle structured region is assigned to disorder in the DisProt-PDB, this is because disorder supercedes structure present in the PDB database.

Statistical analysis methods

True positive rate (TPR): also called the sensitivity, this is the proportion of positive classifications that are actually positive, given by $TPR = \left(\frac{TP}{TP+FN}\right)$.

True negative rate (TNR): also called the specificity, this is the proportion of negative classifications that are actually negative, given by $TNR = \left(\frac{TN}{TN+FP}\right)$.

False positive rate (FPR): also called the fall-out, this is the proportion of positive clas-

sifications that are actually negative, given by $FPR = 1 - TNR$.

False negative rate (FNR): also called the fall-out, this is the proportion of negative classifications that are actually positive, given by $FNR = 1 - TPR$.

Table M1: Confusion matrix (also known as error matrix or matching matrix depending on the context) for the disorder prediction problem. A given residue is experimentally classified as disordered or ordered, and the prediction can either classify a residue as disordered or ordered. This gives rise to four classification scenarios: true positive (TP), false positive (FP), false negative (FN) and true negative (TN).

		Experiment	
		Disordered	Ordered
Predictor	Disordered	TP	FP
	Ordered	FN	TN

Reciever operating characteristic[1] (ROC) and **precision-recall** (PR) are both methods for analyzing the data encoded in the confusion matrix (Table M1) allowing one to construct a point either in ROC or PR space. In ROC space, we plot the TPR vs FPR. Conversely, in PR space we plot the Precision vs Recall (TPR) $\left(\frac{TP}{TP+FP}\right)$. We note that because the Recall and Precision equations lack true negatives PR curves can perform poorly if the underlying dataset is imbalanced with few negatives; the ROC curve is insensitive to class imbalance because it does account for these true negatives.

Precision/Positive predictive value (PPV): the proportion of positive results that are actually true positives $(\frac{TP}{TP+FP})$.

Area under curve (AUC): the numerically integrated (trapezoidal) area under the ROC curve that provides an aggregate measure of performance across all possible classification thresholds.

$\mathbf{F}_{\max}$: the maximum F1 score[2], which is itself the harmonic mean of the precision and recall $\left(2 \cdot \frac{\text{Precision} \cdot \text{Recall}}{\text{Precision}+\text{Recall}}\right)$ and takes into account predictions across the entire sensitivity spec-

trum[3].

Balanced accuracy (BAC): the average of the sensitivity $\left(\frac{TP}{TP+FN}\right)$ and specificity $\left(\frac{TN}{FP+TN}\right)$ that is particularly useful when the underlying classes are imbalanced.

Matthews correlation coefficient[4] (MCC): the Pearson product-moment correlation coefficient between the actual and predicted values. Given explicitly from the confusion matrix (Table M1) as

$$\text{MCC} = \frac{TP \times TN - FP \times FN}{\sqrt{(TP + FP)\,(TP + FN)\,(TN + FP)\,(TN + FN)}}.$$

Unlike the F_1 score and the accuracy the MCC considers all four classes of the confusion matrix making it arguably a superior metric especially when dealing with unbalanced data[5].

Supplemental Figures and Tables

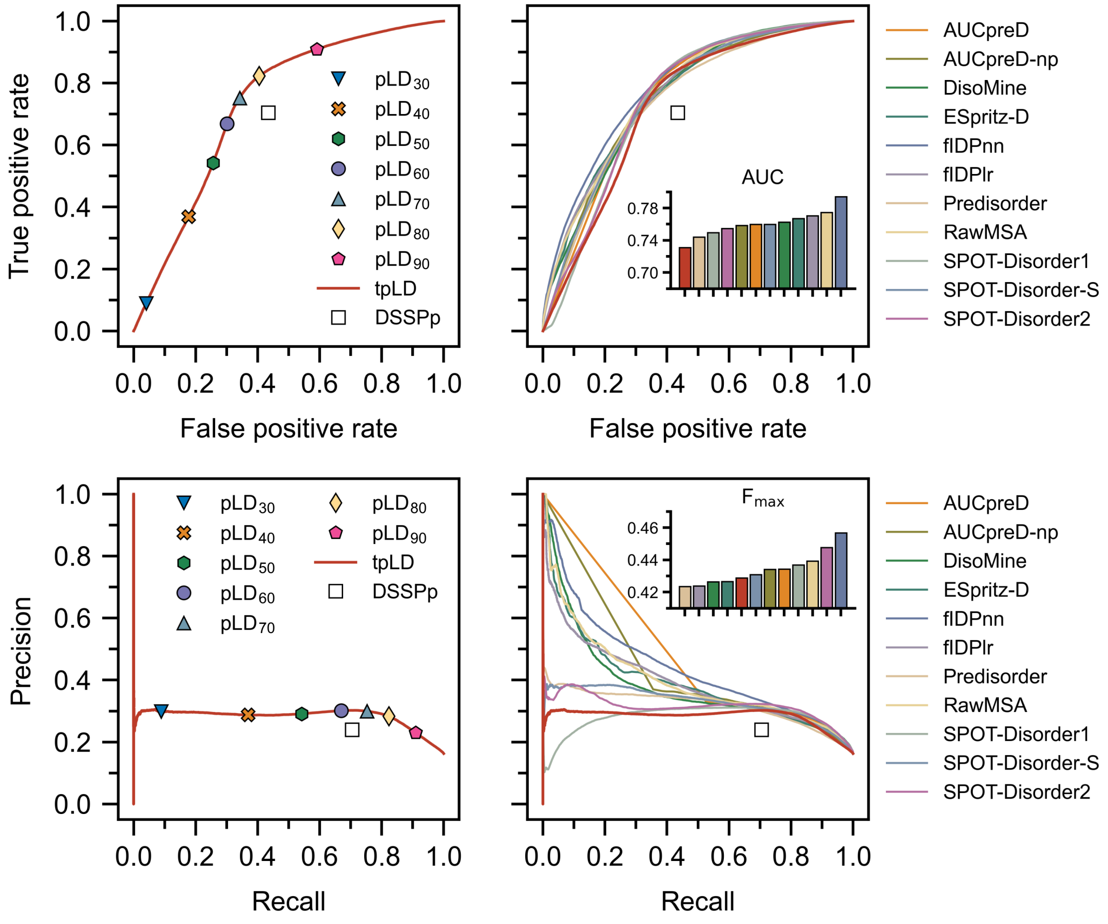

Figure S1: Receiver operating characteristic (ROC) curves (**top**) and precision-recall (**bottom**) are depicted for various predictors calculated per-residue on the DisProt dataset. Note that a ROC curve captures the probability of true and false positives at all thresholds, where an ideal predictor will have an area under the curve (AUC) equal to 1. Further note that a precision-recall curve, captures the trade-off between precision and recall; again, in the ideal case the harmonic mean of the precision and recall (F_{max}) will be equal to 1; bar colors correspond to the legend, red denotes tpLD. In all cases the tpLD (Equation (1) in main text) and various discrete pLD_n predictors are indicated alongside DSSPp. pLDDT is abbreviated pLD for plotting purposes.

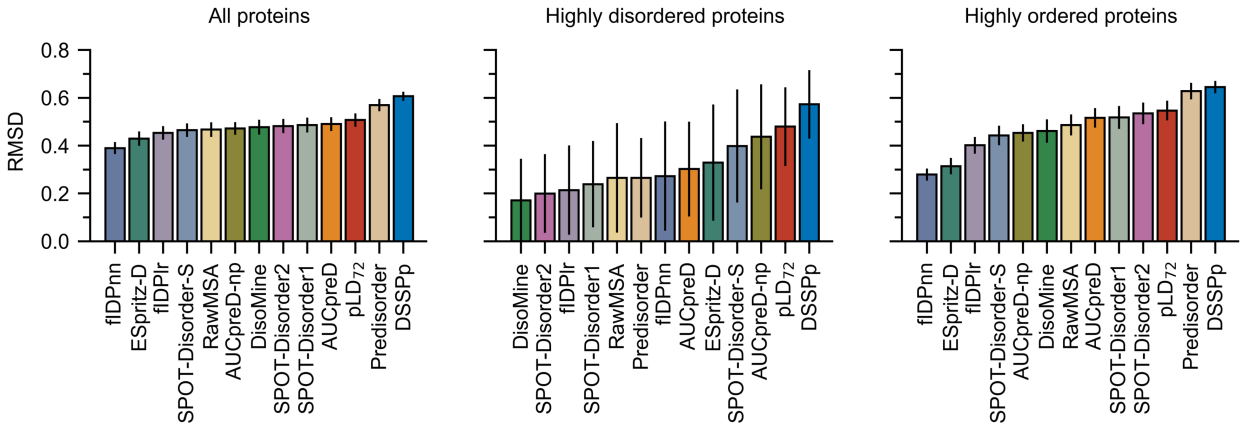

Figure S2: Average RMSD (Equation (2) in main text) values calculated for the DisProt dataset using various prediction methods calculated per-protein. Proteins were assigned to classes depending on disorder content (highly disordered i.e. $> 90\%$ disorder and highly ordered i.e. $< 10\%$ disorder). Bootstrapping, that is, sampling with replacement, was used to compute averages and estimate errors with 10,000 samples of size 60. pLD_{72} resulted in much higher RMSD values than the sequence-based predictors. pLDDT is abbreviated pLD for plotting purposes.

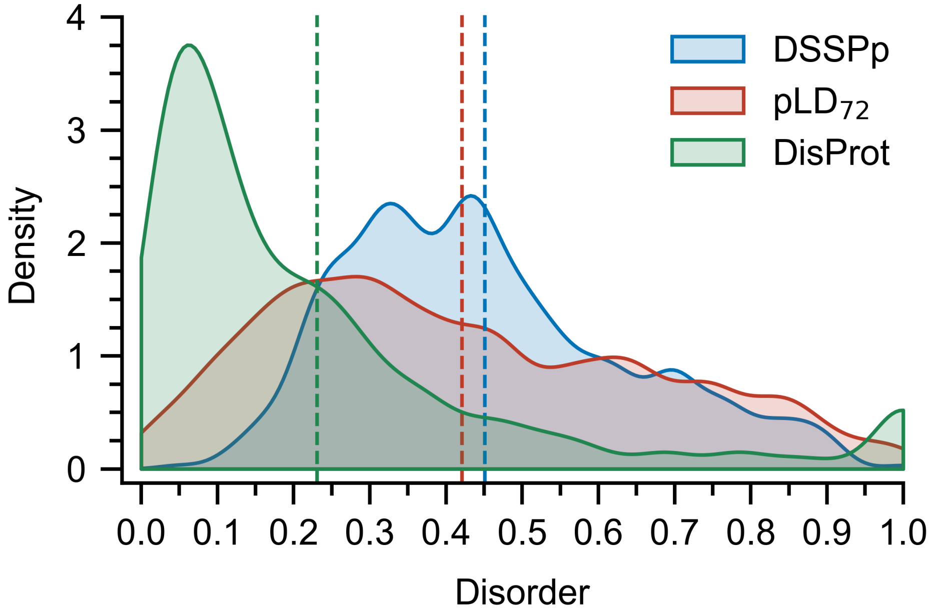

Figure S3: Distribution of disorder content per-protein in the DisProt-PDB and DisProt datasets depicted alongside the distributions predicted by pLD_{72} and DSSPp. Bin-widths were set at 0.5 and bootstrapping, that is, sampling with replacement, was used to compute the distributions and average values (vertical dashed lines) with 10,000 samples of size 60. Poor agreement between DisProt and pLD_{72} is evident. pLDDT is abbreviated pLD for plotting purposes.

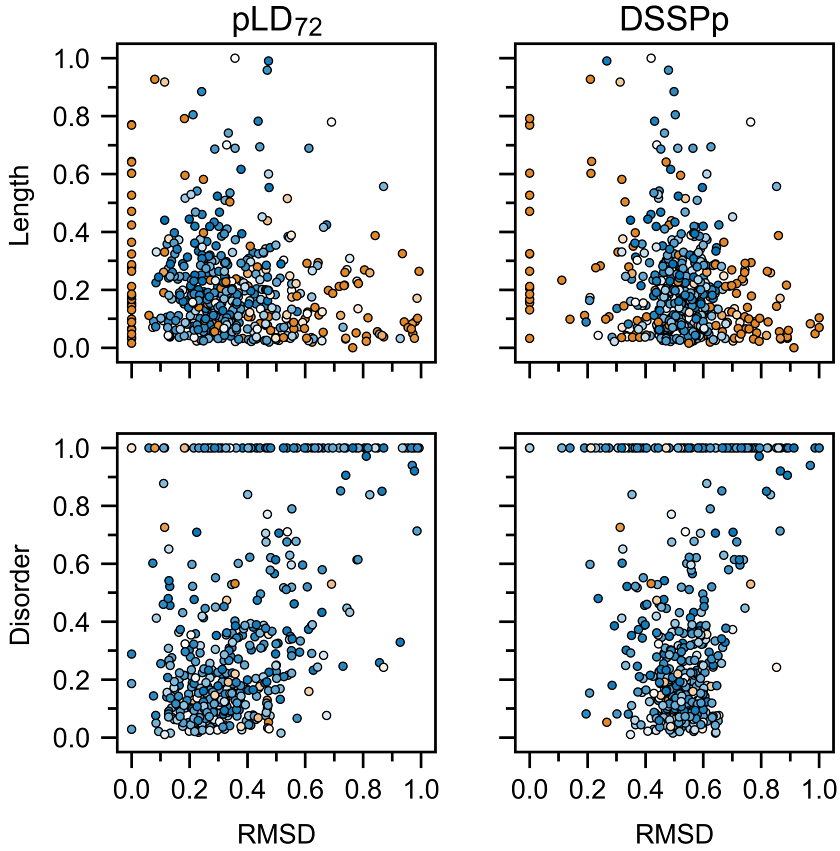

Figure S4: Relationship between protein length (scaled between 0 and 1) and RMSD and protein-wise disorder content and RMSD compared between pLD_{72} and DSSPp on the DisProt-PDB dataset. Colors (0: blue, 0.5: white, 1: orange) indicate disorder content (upper plots) or length (scaled between 0 and 1) (lower plots). Each point represents a protein. pLDDT is abbreviated pLD for plotting purposes.

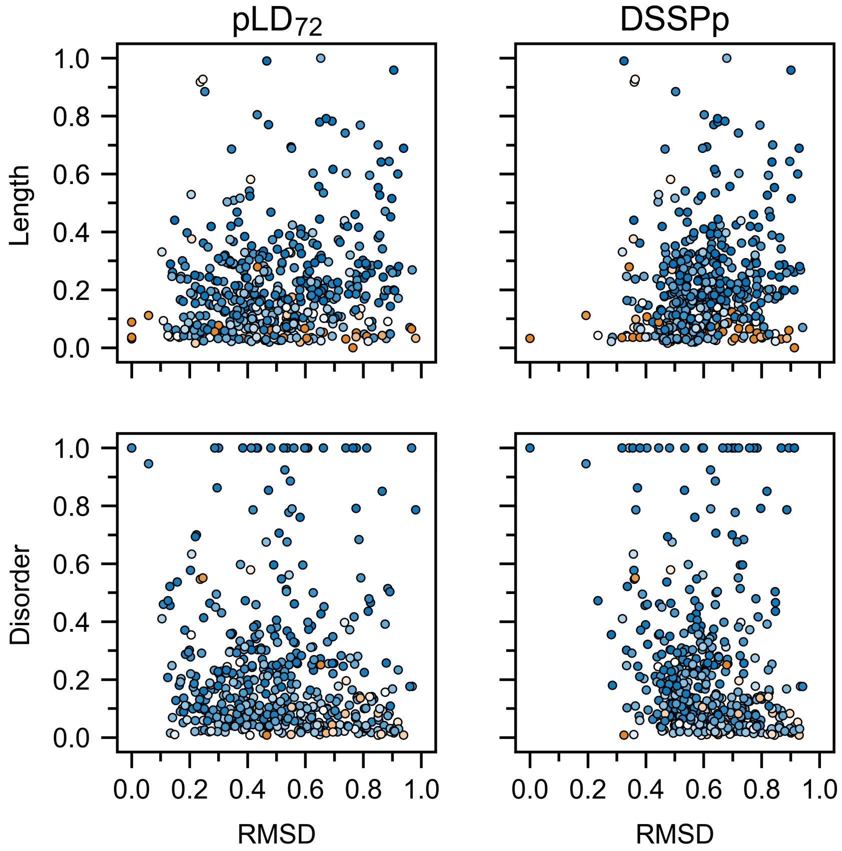

Figure S5: Relationship between protein length (scaled between 0 and 1) and RMSD, and protein-wise disorder content and RMSD compared between pLD_{72} and DSSPp on the DisProt dataset. Colors (0: blue, 0.5: white, 1: orange) indicate disorder content (upper plots) or length (scaled between 0 and 1) (lower plots). Each point represents a protein. pLDDT is abbreviated pLD for plotting purposes.

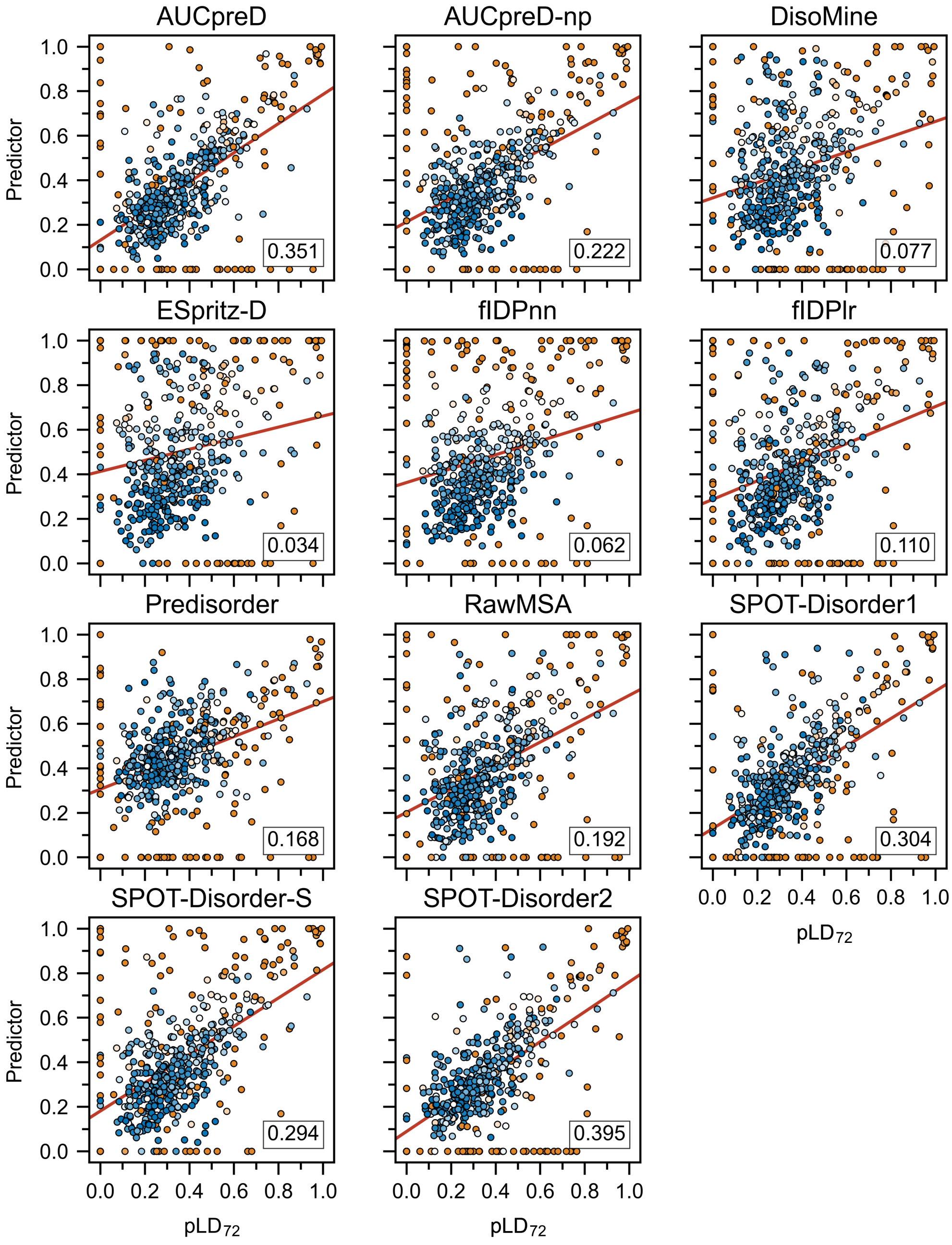

Figure S6: Correlation between conventional predictors and pLD_{72} for predicting protein-wise disorder RMSD on the DisProt-PDB dataset. Colors (0: blue, 0.5: white, 1: orange) indicate protein length (scaled between 0 and 1). Regression lines are colored red and the number in the bottom right is the r^2. Each point represents a protein. pLDDT is abbreviated pLD for plotting purposes.

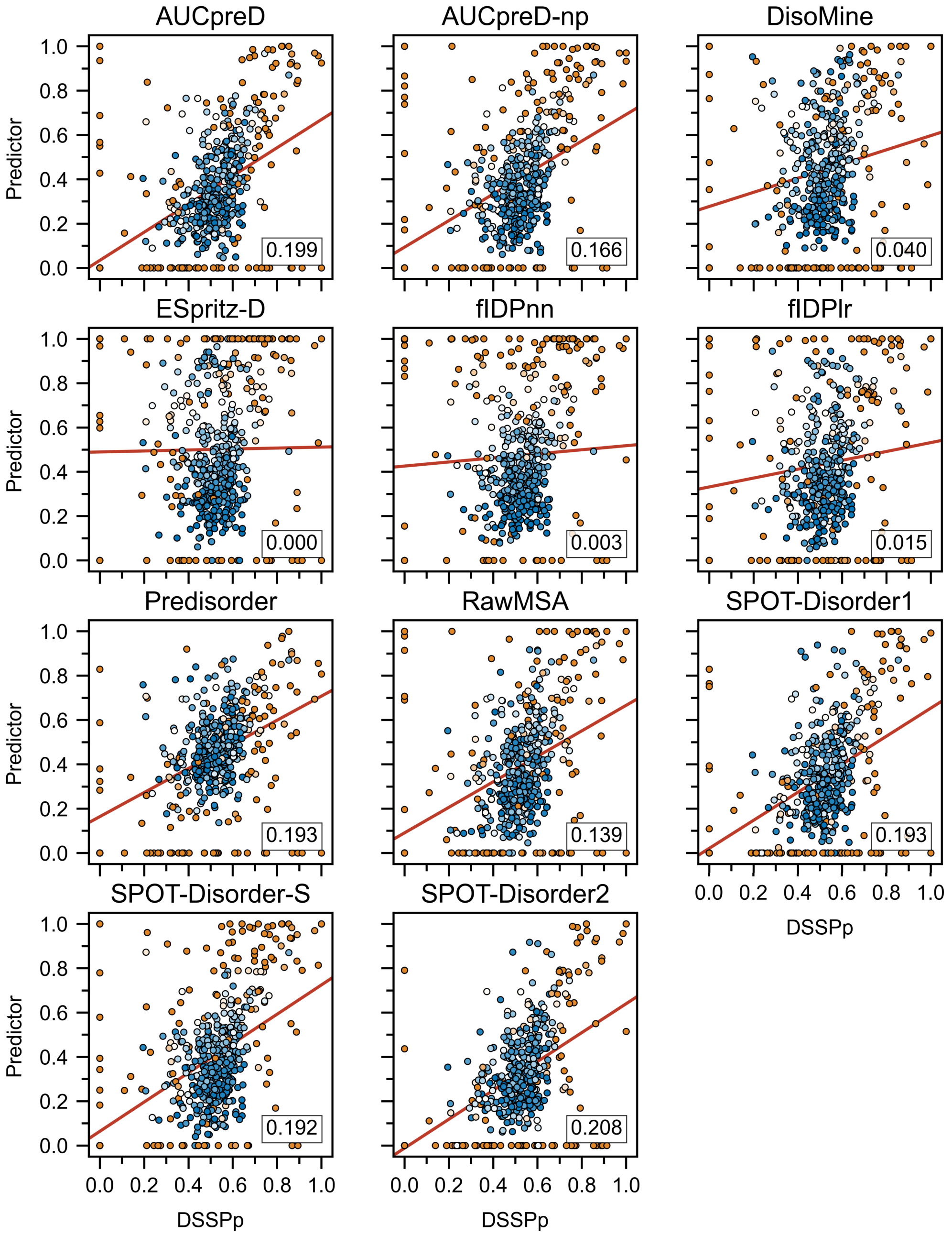

Figure S7: Correlation between conventional predictors and DSSPp for predicting protein-wise disorder RMSD on the DisProt-PDB dataset. Colors (0: blue, 0.5: white, 1: orange) indicate protein length (scaled between 0 and 1). Regression lines are colored red and the number in the bottom right is the r^2. Each point represents a protein.

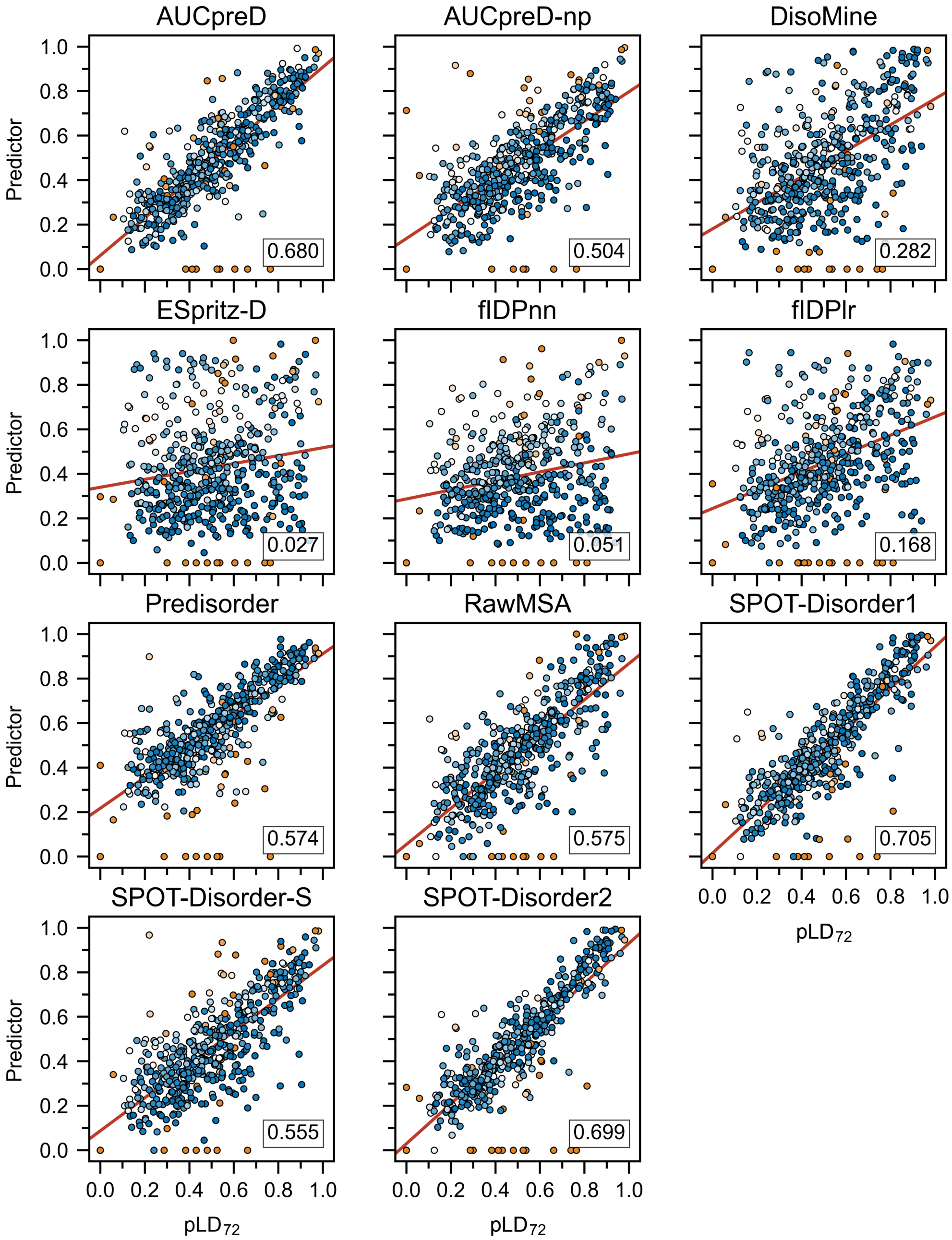

Figure S8: Correlation between conventional predictors and pLD_{72} for predicting protein-wise disorder RMSD on the DisProt dataset. Colors (0: blue, 0.5: white, 1: orange) indicate protein length (scaled between 0 and 1). Regression lines are colored red and the number in the bottom right is the r^2. Each point represents a protein. pLDDT is abbreviated pLD for plotting purposes.

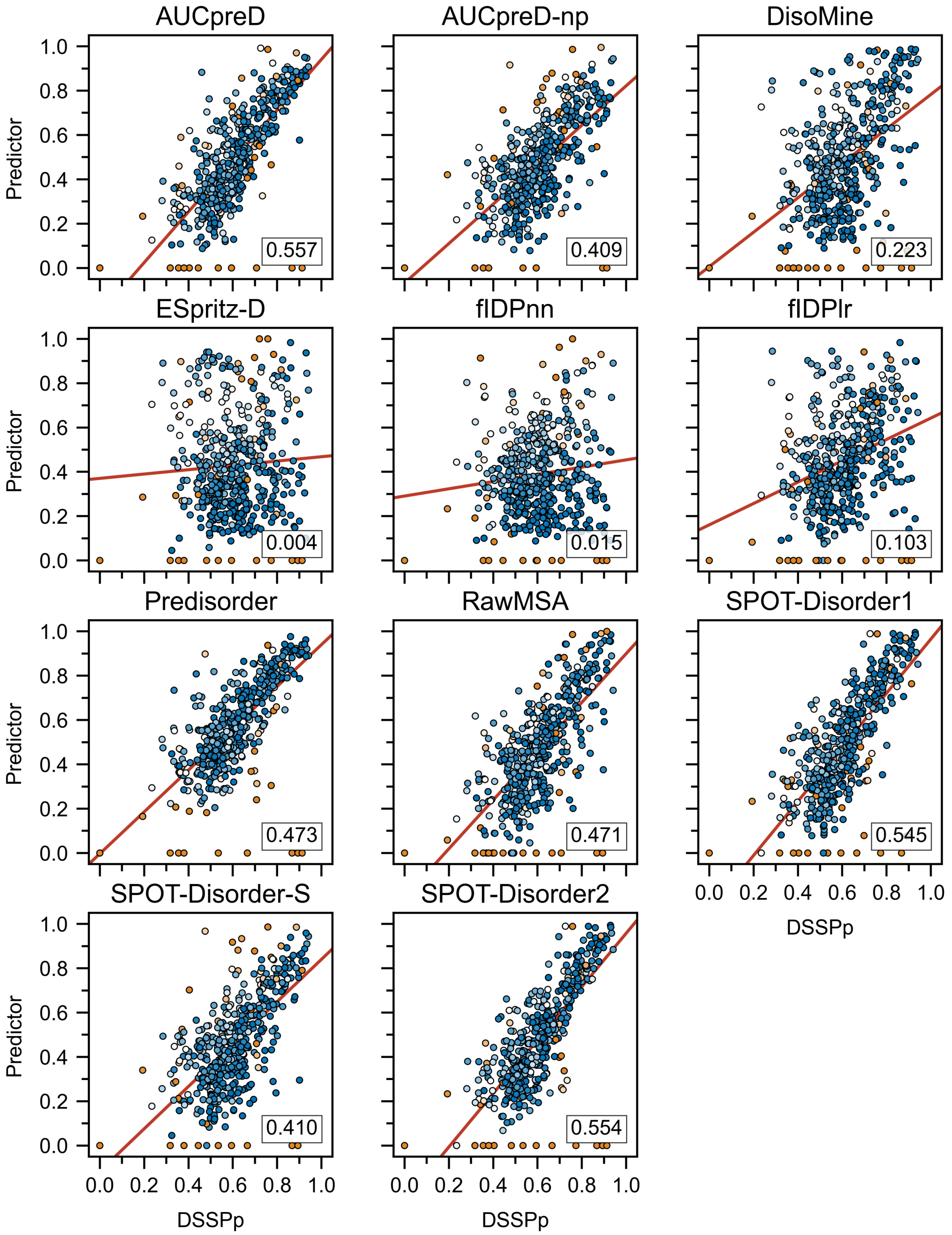

Figure S9: Correlation between conventional predictors and DSSPp for predicting protein-wise disorder RMSD on the DisProt dataset. Colors (0: blue, 0.5: white, 1: orange) indicate protein length (scaled between 0 and 1). Regression lines are colored red and the number in the bottom right is the r^2. Each point represents a protein.

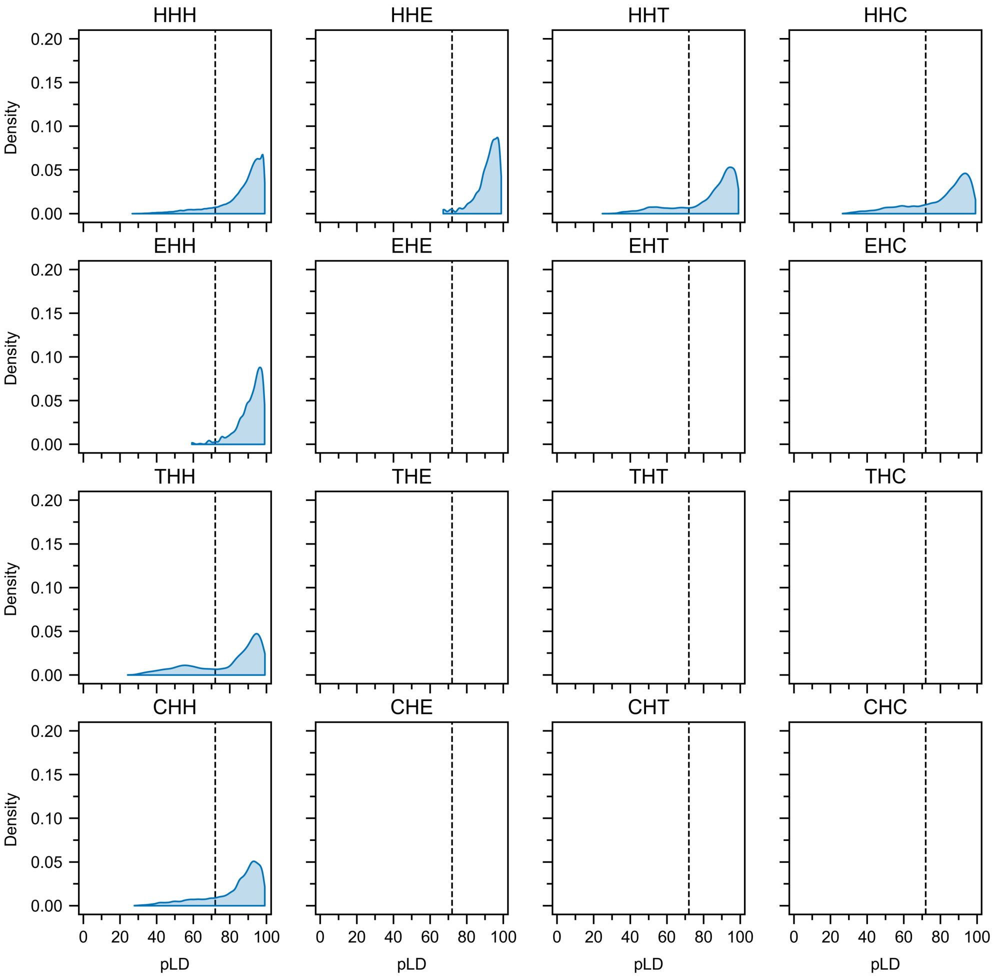

Figure S10: Distributions of pLD values for helical (α-helix, 310-helix and π-helix) secondary structure codons (SSC). Bin-widths were set at 0.5, vertical lines indicate pLD_{72}. pLDDT is abbreviated pLD for plotting purposes.

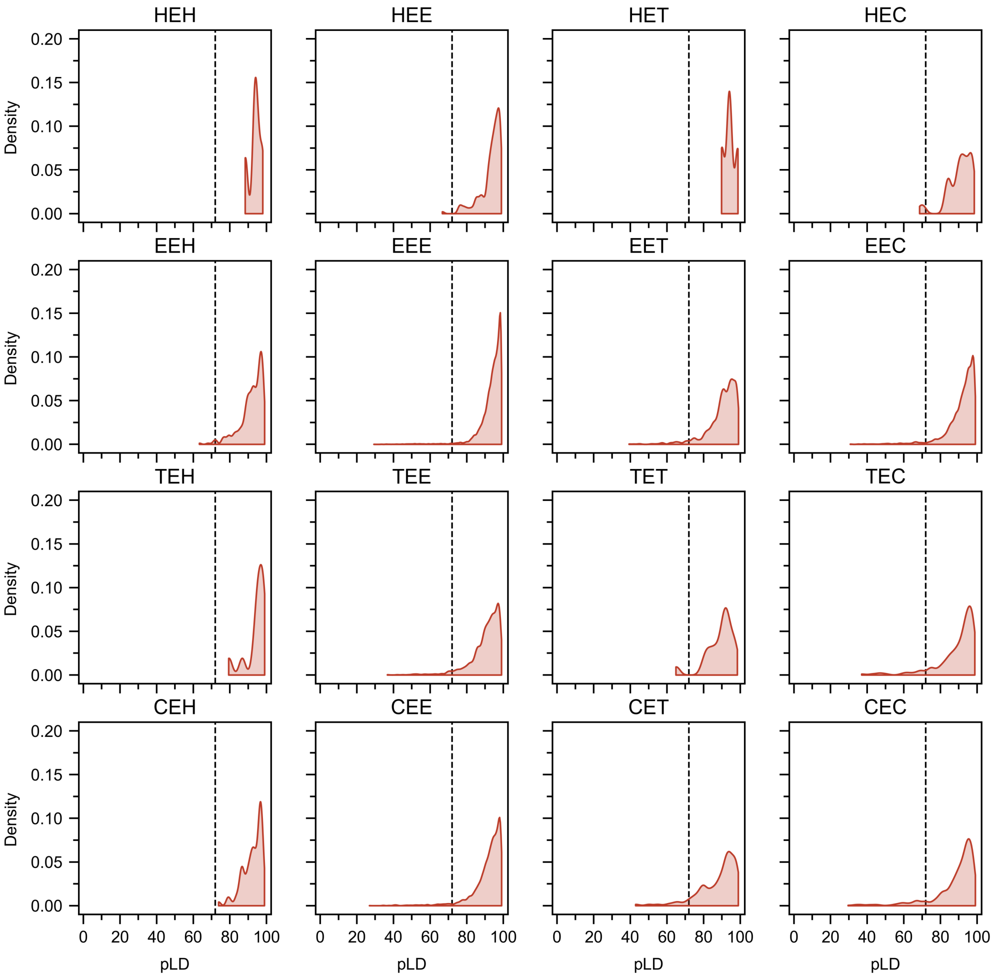

Figure S11: Distributions of pLD values for strand (β-strand and β-bridge) secondary structure codons (SSC). Bin-widths were set at 0.5, vertical lines indicate pLD_{72}. pLDDT is abbreviated pLD for plotting purposes.

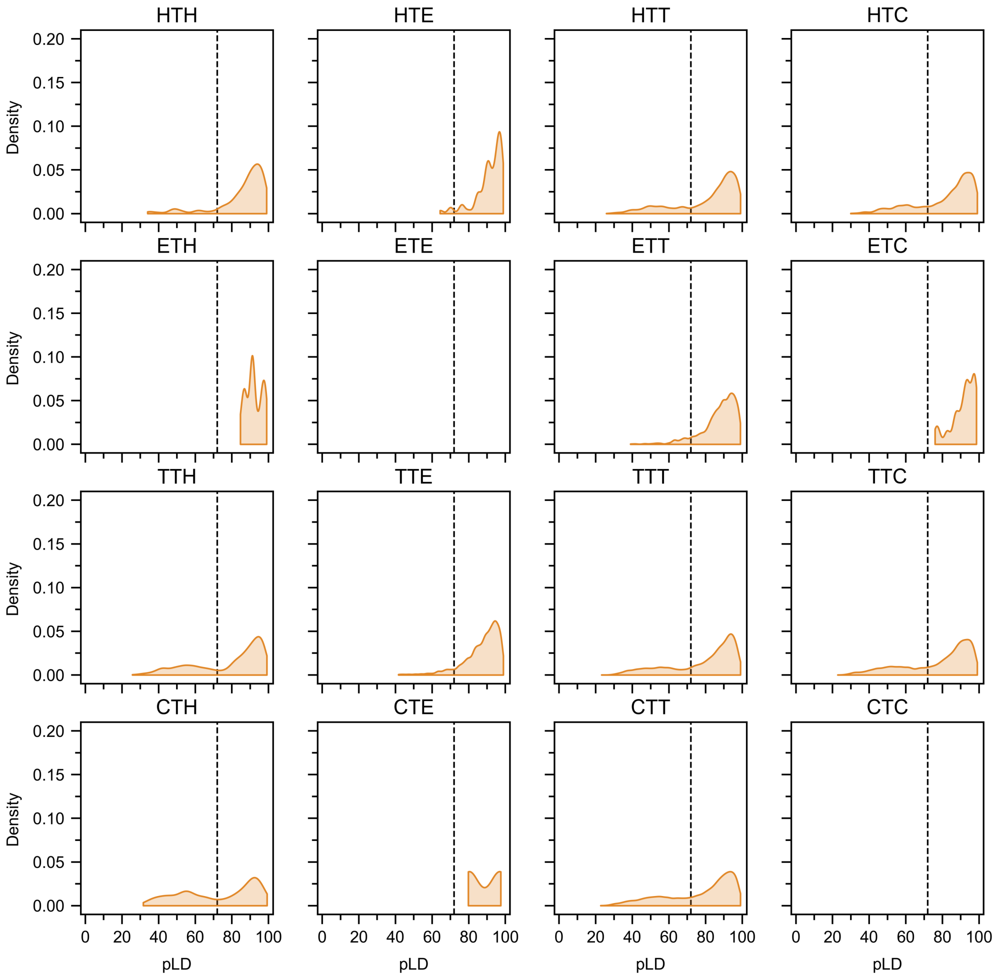

Figure S12: Distributions of pLD values for turn (H-bond stabilized turn) secondary structure codons (SSC). Bin-widths were set at 0.5, vertical lines indicate pLD_{72}. pLDDT is abbreviated pLD for plotting purposes.

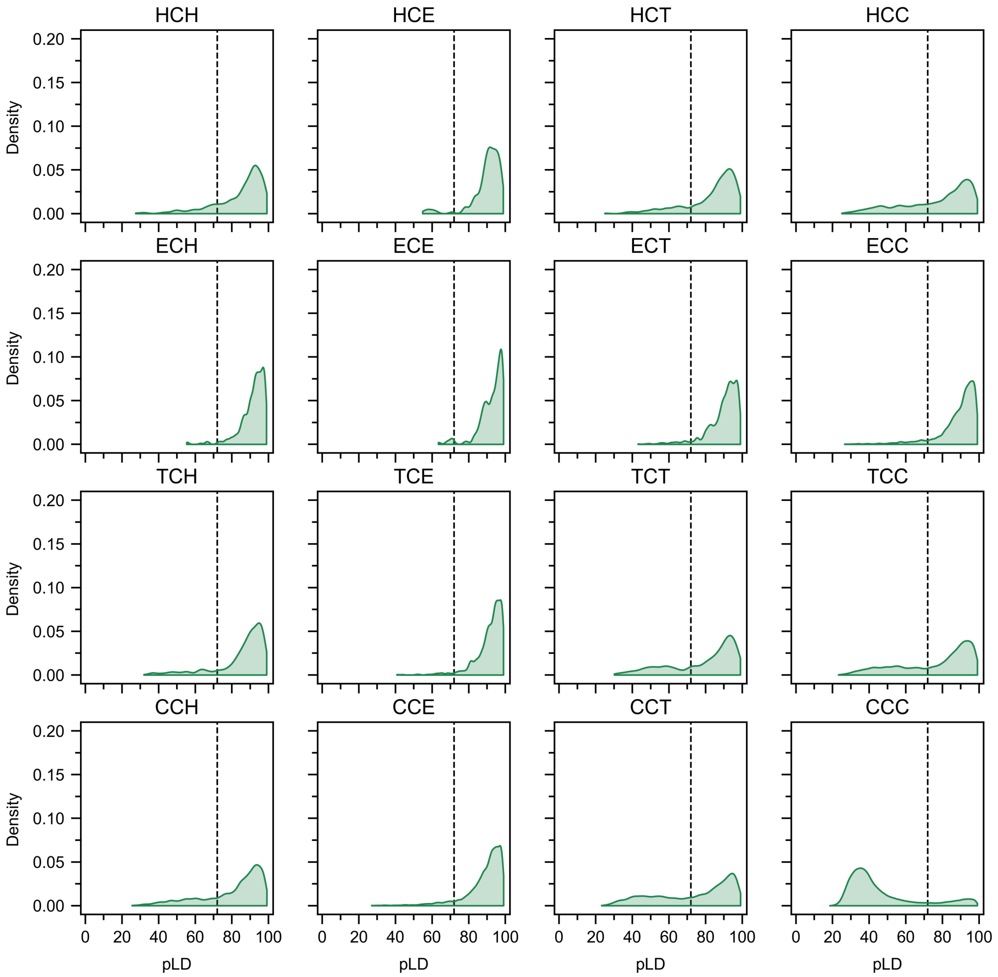

Figure S13: Distributions of pLD values for coil (coil or bend) secondary structure codons (SSC). Bin-widths were set at 0.5, vertical lines indicate pLD_{72}. pLDDT is abbreviated pLD for plotting purposes.

Table S1: Tabulated performance metrics for pLD-based (tpLD and pLD_n) and DSSPp predictors on the DisProt-PDB dataset. Abbreviations: Matthews correlation coefficent (MCC); area under ROC curve (AUC); maximum F1-score (F_{max}); true postivie rate (TPR); false positive rate (FPR); true negative rate (TNR); positive predictive value (PPV); balanced accuracy (BAC); number of proteins covered (COV); predicted Local Distance Difference Test (pLD).

Pred	MCC	AUC	F_{max}	TPR	FPR	TNR	PPV	BAC	COV
DSSPp	0.418	0.724	0.606	0.704	0.257	0.743	0.532	0.724	478
tpLD	N/A	0.905	0.784	N/A	N/A	N/A	N/A	N/A	478
pLD_{30}	0.243	0.544	0.453	0.089	0.002	0.998	0.952	0.544	478
pLD_{32}	0.308	0.569	0.453	0.141	0.003	0.997	0.948	0.569	478
pLD_{34}	0.368	0.598	0.453	0.200	0.005	0.995	0.944	0.598	478
pLD_{36}	0.422	0.626	0.453	0.259	0.007	0.993	0.942	0.626	478
pLD_{38}	0.469	0.654	0.474	0.317	0.009	0.991	0.937	0.654	478
pLD_{40}	0.510	0.679	0.529	0.369	0.011	0.989	0.936	0.679	478
pLD_{42}	0.542	0.700	0.572	0.413	0.013	0.987	0.931	0.700	478
pLD_{44}	0.566	0.717	0.605	0.450	0.015	0.985	0.926	0.717	478
pLD_{46}	0.589	0.734	0.635	0.484	0.017	0.983	0.922	0.734	478
pLD_{48}	0.607	0.747	0.659	0.514	0.020	0.980	0.916	0.747	478
pLD_{50}	0.623	0.760	0.679	0.542	0.022	0.978	0.910	0.760	478
pLD_{52}	0.639	0.722	0.699	0.569	0.025	0.975	0.904	0.722	478
pLD_{54}	0.651	0.783	0.715	0.595	0.029	0.971	0.896	0.783	478
pLD_{56}	0.664	0.794	0.731	0.621	0.032	0.968	0.889	0.794	478
pLD_{58}	0.672	0.805	0.746	0.646	0.036	0.964	0.882	0.805	478
pLD_{60}	0.686	0.815	0.758	0.669	0.040	0.960	0.874	0.815	478
pLD_{62}	0.692	0.822	0.766	0.688	0.045	0.955	0.864	0.822	478
pLD_{64}	0.697	0.828	0.723	0.706	0.050	0.950	0.854	0.828	478
pLD_{66}	0.700	0.834	0.728	0.723	0.056	0.944	0.843	0.834	478
pLD_{68}	0.701	0.838	0.782	0.738	0.062	0.938	0.830	0.838	478
pLD_{70}	0.699	0.841	0.783	0.752	0.070	0.930	0.817	0.841	478
pLD_{72}	0.697	0.844	0.784	0.766	0.079	0.921	0.802	0.844	478
pLD_{74}	0.693	0.846	0.783	0.780	0.088	0.912	0.786	0.846	478
pLD_{76}	0.688	0.847	0.781	0.795	0.100	0.900	0.767	0.847	478
pLD_{78}	0.679	0.847	0.726	0.809	0.114	0.886	0.746	0.847	478
pLD_{80}	0.667	0.846	0.769	0.823	0.132	0.868	0.721	0.846	478
pLD_{82}	0.648	0.841	0.756	0.837	0.156	0.844	0.690	0.841	478
pLD_{84}	0.623	0.833	0.740	0.853	0.187	0.813	0.654	0.833	478
pLD_{86}	0.591	0.820	0.718	0.871	0.230	0.720	0.611	0.820	478
pLD_{88}	0.550	0.801	0.689	0.889	0.287	0.713	0.562	0.801	478
pLD_{90}	0.500	0.725	0.655	0.909	0.360	0.640	0.511	0.725	478

Table S2: Tabulated performance metrics for pLD-based (tpLD and pLD_n) and DSSPp predictors on the DisProt dataset. Abbreviations: Matthews correlation coefficent (MCC); area under ROC curve (AUC); maximum F1-score (F_{max}); true postivie rate (TPR); false positive rate (FPR); true negative rate (TNR); positive predictive value (PPV); balanced accuracy (BAC); number of proteins covered (COV); predicted Local Distance Difference Test (pLD).

Pred	MCC	AUC	F_{max}	TPR	FPR	TNR	PPV	BAC	COV
DSSPp	0.199	0.635	0.357	0.704	0.434	0.566	0.240	0.635	478
tpLD	NA	0.731	0.429	N/A	N/A	N/A	N/A	N/A	478
pLD_{30}	0.083	0.524	0.280	0.089	0.041	0.959	0.299	0.524	478
pLD_{32}	0.105	0.538	0.280	0.141	0.065	0.935	0.296	0.538	478
pLD_{34}	0.127	0.554	0.280	0.200	0.093	0.907	0.296	0.554	478
pLD_{36}	0.144	0.569	0.280	0.259	0.122	0.878	0.292	0.569	478
pLD_{38}	0.160	0.583	0.303	0.317	0.151	0.849	0.290	0.583	478
pLD_{40}	0.174	0.596	0.323	0.369	0.172	0.823	0.288	0.596	478
pLD_{42}	0.186	0.607	0.339	0.413	0.199	0.801	0.287	0.607	478
pLD_{44}	0.197	0.616	0.350	0.450	0.217	0.783	0.287	0.616	478
pLD_{46}	0.209	0.626	0.361	0.484	0.232	0.768	0.288	0.626	478
pLD_{48}	0.219	0.635	0.370	0.514	0.245	0.755	0.290	0.635	478
pLD_{50}	0.229	0.643	0.379	0.542	0.256	0.744	0.291	0.643	478
pLD_{52}	0.241	0.651	0.387	0.569	0.266	0.734	0.293	0.651	478
pLD_{54}	0.251	0.660	0.395	0.595	0.276	0.724	0.295	0.660	478
pLD_{56}	0.262	0.668	0.402	0.621	0.285	0.715	0.298	0.668	478
pLD_{58}	0.273	0.672	0.410	0.646	0.293	0.707	0.300	0.672	478
pLD_{60}	0.283	0.684	0.416	0.669	0.301	0.699	0.301	0.684	478
pLD_{62}	0.290	0.690	0.420	0.688	0.309	0.691	0.302	0.690	478
pLD_{64}	0.296	0.695	0.423	0.706	0.317	0.683	0.302	0.695	478
pLD_{66}	0.302	0.699	0.426	0.723	0.325	0.675	0.302	0.699	478
pLD_{68}	0.305	0.703	0.428	0.738	0.333	0.667	0.301	0.703	478
pLD_{70}	0.308	0.705	0.428	0.752	0.342	0.658	0.299	0.705	478
pLD_{72}	0.310	0.707	0.429	0.766	0.351	0.649	0.298	0.707	478
pLD_{74}	0.312	0.709	0.428	0.780	0.362	0.638	0.295	0.709	478
pLD_{76}	0.313	0.710	0.427	0.795	0.374	0.626	0.292	0.710	478
pLD_{78}	0.312	0.710	0.425	0.809	0.388	0.612	0.288	0.710	478
pLD_{80}	0.310	0.709	0.422	0.823	0.405	0.595	0.283	0.709	478
pLD_{82}	0.303	0.705	0.415	0.837	0.426	0.574	0.276	0.705	478
pLD_{84}	0.294	0.699	0.407	0.853	0.454	0.546	0.267	0.699	478
pLD_{86}	0.282	0.690	0.396	0.871	0.490	0.510	0.256	0.690	478
pLD_{88}	0.266	0.672	0.383	0.889	0.535	0.465	0.244	0.672	478
pLD_{90}	0.245	0.659	0.367	0.909	0.591	0.409	0.230	0.659	478

Table S3: Tabulated performance metrics for conventional predictors on the DisProt-PDB dataset. Abbreviations: Matthews correlation coefficent (MCC); area under ROC curve (AUC); maximum F1-score (F_{max}); true postivie rate (TPR); false positive rate (FPR); true negative rate (TNR); positive predictive value (PPV); balanced accuracy (BAC); number of proteins covered (COV).

Pred	MCC	AUC	F_{max}	TPR	FPR	TNR	PPV	BAC	COV
AUCpreD	0.673	0.909	0.768	0.709	0.065	0.935	0.818	0.822	475
AUCpreD-np	0.610	0.886	0.731	0.589	0.047	0.953	0.838	0.721	475
DisoMine	0.509	0.858	0.688	0.569	0.095	0.905	0.712	0.737	475
ESpritz-D	0.397	0.854	0.679	0.333	0.042	0.958	0.765	0.645	475
fIDPnn	0.390	0.866	0.689	0.253	0.015	0.985	0.876	0.619	475
fIDPlr	0.484	0.851	0.666	0.467	0.059	0.941	0.765	0.704	475
Predisorder	0.557	0.876	0.723	0.805	0.210	0.709	0.614	0.798	475
RawMSA	0.614	0.895	0.746	0.659	0.072	0.923	0.781	0.791	474
SPOT-Disorder1	0.692	0.917	0.783	0.752	0.074	0.926	0.807	0.839	475
SPOT-Disorder-S	0.618	0.896	0.744	0.565	0.033	0.967	0.875	0.766	475
SPOT-Disorder2	0.698	0.917	0.780	0.760	0.073	0.927	0.801	0.843	451

Table S4: Tabulated performance metrics for conventional predictors on the DisProt dataset. Abbreviations: Matthews correlation coefficent (MCC); area under ROC curve (AUC); maximum F1-score (F_{max}); true postivie rate (TPR); false positive rate (FPR); true negative rate (TNR); positive predictive value (PPV); balanced accuracy (BAC); number of proteins covered (COV).

Pred	MCC	AUC	F_{max}	TPR	FPR	TNR	PPV	BAC	COV
AUCpreD	0.311	0.760	0.434	0.709	0.302	0.698	0.313	0.703	475
AUCpreD-np	0.299	0.758	0.434	0.589	0.224	0.726	0.338	0.682	475
DisoMine	0.274	0.763	0.426	0.569	0.233	0.767	0.321	0.668	475
ESpritz-D	0.268	0.767	0.427	0.333	0.090	0.910	0.419	0.622	475
fIDPnn	0.284	0.794	0.457	0.253	0.045	0.955	0.521	0.604	475
fIDPlr	0.287	0.720	0.424	0.467	0.153	0.847	0.372	0.657	475
Predisorder	0.280	0.744	0.423	0.805	0.426	0.574	0.268	0.690	475
RawMSA	0.306	0.725	0.439	0.657	0.269	0.731	0.322	0.696	474
SPOT-Disorder1	0.317	0.749	0.437	0.752	0.331	0.669	0.306	0.711	475
SPOT-Disorder-S	0.282	0.760	0.431	0.565	0.222	0.728	0.330	0.672	475
SPOT-Disorder2	0.329	0.755	0.448	0.437	0.391	0.609	0.191	0.716	451

References

(1) Fawcett, T. An introduction to ROC analysis. *Pattern Recognit. Lett.* **2006**, *27*, 861–874.

(2) Rijsbergen, C. J. *Information retrieval*; Butterworth-Heinemann: Oxford, United Kingdom, 1979.

(3) Radivojac, P.; Clark, W. T.; Oron, T. R.; Schnoes, A. M.; Wittkop, T.; Sokolov, A.; Graim, K.; Funk, C.; Verspoor, K.; Ben-Hur, A.; Pandey, G.; Yunes, J. M.; Talwalkar, A. S.; Repo, S.; Souza, M. L.; Piovesan, D.; Casadio, R.; Wang, Z.; Cheng, J.; Fang, H.; Gough, J.; Koskinen, P.; Törönen, P.; Nokso-Koivisto, J.; Holm, L.; Cozzetto, D.; Buchan, D. W. A.; Bryson, K.; Jones, D. T.; Limaye, B.; Inamdar, H.; Datta, A.; Manjari, S. K.; Joshi, R.; Chitale, M.; Kihara, D.; Lisewski, A. M.; Erdin, S.; Venner, E.; Lichtarge, O.; Rentzsch, R.; Yang, H.; Romero, A. E.; Bhat, P.; Paccanaro, A.; Hamp, T.; Kaßner, R.; Seemayer, S.; Vicedo, E.; Schaefer, C.; Achten, D.; Auer, F.; Boehm, A.; Braun, T.; Hecht, M.; Heron, M.; Hönigschmid, P.; Hopf, T. A.; Kaufmann, S.; Kiening, M.; Krompass, D.; Landerer, C.; Mahlich, Y.; Roos, M.; Björne, J.; Salakoski, T.; Wong, A.; Shatkay, H.; Gatzmann, F.; Sommer, I.; Wass, M. N.; Sternberg, M. J. E.; Škunca, N.; Supek, F.; Bošnjak, M.; Panov, P.; Džeroski, S.; Šmuc, T.; Kourmpetis, Y. A. I.; van Dijk, A. D. J.; ter Braak, C. J. F.; Zhou, Y.; Gong, Q.; Dong, X.; Tian, W.; Falda, M.; Fontana, P.; Lavezzo, E.; Camillo, B. D.; Toppo, S.; Lan, L.; Djuric, N.; Guo, Y.; Vucetic, S.; Bairoch, A.; Linial, M.; Babbitt, P. C.; Brenner, S. E.; Orengo, C.; Rost, B.; Mooney, S. D.; Friedberg, I. A large-scale evaluation of computational protein function prediction. *Nat. Methods* **2013**, *10*, 221–227.

(4) Matthews, B. W. Comparison of the predicted and observed secondary structure of T4 phage lysozyme. *Biochim. Biophys. Acta* **1975**, *405*, 442–451.

(5) Chicco, D.; Jurman, G. The advantages of the Matthews correlation coefficient (MCC)

over F1 score and accuracy in binary classification evaluation. *BMC Genomics* **2020**, *21*.
